# Event and Comment

**Published:** 1907-12-15

**Authors:** 


### DENTAL REGISTER.

Vol. LXI. December 15, 1907. No. 12.

### EVENT AND COMMENT.

### Tooth Roots.

For the last twenty-five years, it would seem, the dental profession has given the larger part of its time and thought to the saving or repairing of the crowns of the teeth, and consequently has attached an undue importance to that portion of the tooth, to the neglect of the root more particularly. It is true we have been conscious all this while that no crown, however perfectly formed and preserved, would be of much value without a root to stand it on. The propoganda for oral prophylaxis so strenuously carried on by Dr. D. D. Smith for the salvation of the roots of the teeth by a systematic method of treatment, has directed our attention to the value of the roots of the teeth, so much so that their conservation is fast coming to be one of the greatest and best advances made in dentistry in the past decade. The wonder is that we have allowed the condition of the gums, brought about by deposits on the roots of the teeth, to become so universally diseased in many cases as to seriously threaten the loss of entire dentures, without becoming more concerned than we have been. A case came to our attention recently where several teeth had been lost, and the patient wished bridgework inserted to supply the lost teeth. An examination revealed the fact that practically every remaining tooth in the mouth was affected with pyorrhea, and most of them were so loose that the occlusion was fast shifting them to serious malocclusion.

There was no visible deposit of consequence, but a careful exploration disclosed pockets varying from one-third to two-thirds of the length of the roots. The patient had no knowledge of this condition and the dentist who expected to construct the bridge work seemed never to have suspected that there was anything to be done to prepare the mouth for bridgework. However skillfully bridge work may have been made for such a mouth, it must have proved an inevitable failure, and in a short time. Another case came under our care recently which seemed to require only a filling to place the mouth in good condition. A small deposit of c-al culus on the buccal surface of the upper first molar called for attention, which led to the fact that almost every tooth in the mouth had serious deposits secretly lodged under the gum and without producing a sign of irritation of the gum or any suppuration. Three sittings of an hour and a half each spent on that mouth were necessary to put the mouth in a wholesome condition. A few days only after the treatment showed such a marked improvement in the condition of the gums that the patient noticed it, and is delighted with the feeling of comfort he now enjoys, The gums have settled down close to the roots, and are hard and of a beautiful pink color, and the patient brushes his teeth with a good stiff brush as much as he likes with no discomfort.

In all patients, particularly in those past the age of thirty years, the roots of the teeth need constant supervision. While we are looking for cavities to fill and crowns and bridges to make, don't forget to carefully examine the root conditions. If the roots fail, what use will all our beautiful crowns and fillings be? The fact is, the props will have been knocked out, and the entire dental structure will fall as a mass of ruins. A new field of service is here opening that will require our highest skill to successfully meet, and heroic devotion to our professional instinct to take up, as it will require much tedious labor, and the remuneration may not always compensate. But we must do it.

